# Neuroprotection against 6-OHDA toxicity in PC12 cells and mice through the Nrf2 pathway by a sesquiterpenoid from *Tussilago farfara*

**DOI:** 10.1016/j.redox.2018.05.015

**Published:** 2018-06-01

**Authors:** Joohee Lee, Kwangho Song, Eugene Huh, Myung Sook Oh, Yeong Shik Kim

**Affiliations:** a Natural Products Research Institute, College of Pharmacy, Seoul National University, Seoul 08826, South Korea; b Department of Medical Science of Meridian, Graduate School, Kyung Hee University, Seoul 02447, South Korea; c Department of Life and Nanopharmaceutical Sciences, Graduate School and Kyung Hee East-West Pharmaceutical Research Institute, Kyung Hee University, Seoul 02447, South Korea

**Keywords:** 6-OHDA, 6-hydroxydopamine, Act. D, actinomycin D, AD, Alzheimer's disease, APO, apomorphine, ARE, antioxidant response element, CHX, cycloheximide, DAT, dopamine transporter, DTT, dithiothreitol, ECN, 7β-(3-ethyl-*cis*-crotonoyloxy)-1α-(2-methylbutyryloxy)-3,14-dehydro-*Z*-notonipetranone, ERK1/2, extracellular signal regulated protein kinase 1/2, HO-1, heme oxygenase-1, H_2_O_2_, hydrogen peroxide, Keap1, Kelch-like ECH-associated protein 1, LPS, lipopolysaccharide, MAPKs, mitogen-activated protein kinases, NAC, *N*-acetylcysteine, Nrf2, nuclear factor-E2-related factor 2, PD, Parkinson's disease, PI3K, phosphoinositide 3-kinase, SN, substantia nigra, SnPP, tin protoporphyrin IX, ST, striatum, Neuroprotection, Neurodegeneration, Nrf2, Heme oxygenase-1, *Tussilago farfara*

## Abstract

Oxidative stress plays a key role in neurodegenerative diseases such as Alzheimer's and Parkinson's diseases. Therefore, the nuclear factor-E2-related factor 2 (Nrf2), a key regulator of the antioxidative response, is considered to be important as a therapeutic target for neurodegenerative diseases. We investigated the underlying mechanism of Nrf2-mediated neuroprotective effects against oxidative stress in the PC12 cell line by 7β-(3-ethyl-*cis*-crotonoyloxy)-1α-(2-methylbutyryloxy)-3,14-dehydro-*Z*-notonipetranone (ECN), one of the sesquiterpenoids in Farfarae Flos. Pretreatment of PC12 cells with ECN had a protective effect against hydrogen peroxide (H_2_O_2_)- or 6-hydroxydopamine (6-OHDA)-induced cytotoxicity. ECN upregulated the ARE-luciferase activity and induced the mRNA expression of Nrf2 and antioxidant enzyme heme oxygenase-1 (HO-1). Knockdown of Nrf2 by small, interfering RNA (siRNA) abrogated the upregulation of HO-1, indicating that ECN had induced HO-1 *via* the Nrf2 pathway. Pretreatment with the thiol reducing agents, *N*-acetylcysteine (NAC) or dithiothreitol (DTT), attenuated Nrf2 activation and HO-1 expression. However, the non-thiol reducing antioxidant, Trolox, failed to inhibit HO-1 induction by ECN. These results suggest that ECN may directly interact with Kelch-like ECH-associated protein 1 (Keap1) and modify critical cysteine thiols present in the proteins responsible for Nrf2-mediated upregulation of HO-1. In a 6-OHDA-induced mouse model of PD, administration of ECN ameliorated motor impairments and dopaminergic neuronal damage. Taken together, ECN exerts neuroprotective effects by activating the Nrf2/HO-1 signaling pathway in both PC12 cells and mice. Thus, ECN, as an Nrf2 activator, could be an attractive therapeutic candidate for the neuroprotection or treatment of neurodegenerative diseases.

## Highlights

• ECN protects PC12 cells against H_2_O_2_- or 6-OHDA-induced cell damage.
• ECN increases HO-1 expression *via* the nuclear translocation and ARE binding of Nrf2.
• Activation of the Nrf2/HO-1 pathway contributes to the protective properties of ECN.
• ECN ameliorates 6-OHDA-induced motor impairments in mice.
• ECN prevents 6-OHDA-induced loss of dopaminergic neurons in the mouse brain.

## Graphical abstract

**Figure:**
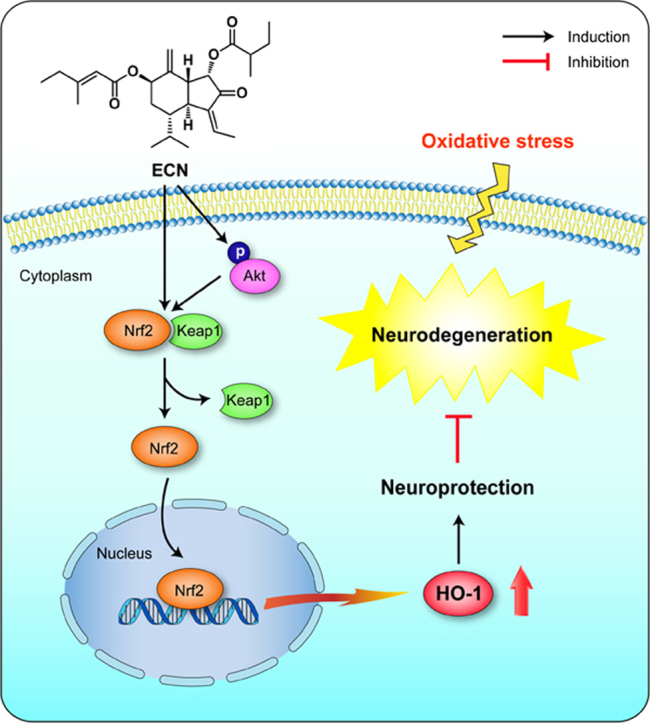

## 1 Introduction

Neurodegenerative diseases, such as Alzheimer's disease (AD), Parkinson's disease (PD), and amyotrophic lateral sclerosis (ALS), are characterized by the progressive dysfunction and loss of structure or function of neurons in the central nervous system [1]. Although the exact cause of each disease remains unclear, many lines of evidence suggest that oxidative stress play a key role in the pathogenesis of neurodegenerative diseases [1], [2]. In the brain, reactive oxygen species (ROS), which cause oxidative stress, are mainly generated by dopamine metabolism, mitochondrial dysfunction, aging, and neuroinflammation. Several proteins related to the pathogenesis of AD and PD, such as amyloid-β peptide (Aβ), amyloid precursor protein (APP), α-synuclein, parkin, PTEN-induced kinase1 (PINK1), DJ-1, and leucine-rich repeat kinase (LRRK2) are also associated with oxidative stress and mitochondrial dysfunction [3], [4]. In this regard, modulation of oxidative stress can be an effective pharmacological strategy for the prevention or treatment of neurodegenerative disorders.

Nuclear factor erythroid 2-related factor 2 (Nrf2) is an essential transcription factor that regulates antioxidant defense genes in maintaining cellular homeostasis. Under normal conditions, Nrf2 remains inactive in the cytoplasm, by forming a complex with its inhibitory protein Kelch-like ECH-associated protein 1 (Keap1), which promotes ubiquitination and the eventual degradation of Nrf2. Under stress conditions, Nrf2 is released from Keap1, translocates to the nucleus and binds to the antioxidant response element (ARE) in the promoter region of several cytoprotective genes, such as heme oxygenase-1 (HO-1) and NAD(P)H:quinone oxidoreductase 1 (NQO1) [5], [6]. The Nrf2 signaling pathway has been reported to be closely related to neurodegenerative diseases [5]. Nrf2 activation in astrocyte mediates neuroprotection against 1-methyl-4-phenyl-1,2,3,6-tetrahydropyridine (MPTP)-induced neurotoxicity PD model in mice [7], and electrophilic compounds show protective effects both *in vitro* and *in vivo* against neuronal degeneration by activating the Keap1/Nrf2/HO-1 pathway [8]. Upregulation of HO-1, one of the target genes induced by Nrf2, plays a key role in neurodegenerative damage associated with AD and PD [9]. Therefore, the Nrf2/HO-1 pathway is a beneficial therapeutic target in the protection or treatment of neurodegenerative diseases.

Phytochemicals are potent antioxidants and act as activators of Nrf2 inducing phase II detoxification enzymes. Terpenoids, including mono-, sesqui-, di-, and triterpenoids, induce Nrf2 through the Michael reaction of reactive cysteine residues on the Keap1 protein. Because of this common feature, various terpenoids have been reported to possess protective effects [10], [11]. A previous study revealed that several sesquiterpenoids isolated from the buds of *Tussilago farfara*, including 7β-(3-ethyl-*cis*-crotonoyloxy)-1α-(2-methylbutyryloxy)-3,14-dehydro-*Z*-notonipetranone (ECN), have anti-inflammatory actions in activated microglia and cytoprotective effects against LPS-induced neuronal cell death [12]. However, the Nrf2-mediated neuroprotective properties of ECN against oxidative stress and *in vivo* studies on the 6-OHDA-induced neurotoxicity in mice have yet to be elucidated. The objective of this study was thus to investigate cytoprotective activities against cell damage induced by oxidative stress and underlying molecular mechanisms of ECN. The potency of ECN to activate Nrf2 and induce HO-1 was also identified. In addition, we aimed to determine whether ECN exerted any protective effects in an animal experimental model of neurodegeneration.

## 2 Materials and methods

### 2.1 Materials and reagents

ECN was isolated from dried buds of *Tussilago farfara* and identified, as previously reported by our group [13]. Fetal bovine serum (FBS), penicillin, and streptomycin were purchased from GenDepot (Barker, TX, USA). Horse serum (HS) was the product of GIBCO BRL (Grand Island, NY, USA). Ham's F-12K, Dulbecco's phosphate buffered saline, 3-(4,5-dimethylthiazol-2-yl)-2,5-diphenyltetrazolium bromide (MTT), 6-hydroxydopamine (6-OHDA), cycloheximide, actinomycin D, 2′,7′-dichlorofluorescein diacetate (DCF-DA), dithiothreitol (DTT), *N*-acetyl-L-cysteine (NAC), LY294002, U0126, SB203580, SP600125, protease inhibitor cocktail, paraformaldehyde (PFA), diaminobenzidine (DAB), sucrose, apomorphine (APO), and tribromoethanol (TBE) were purchased from Sigma-Aldrich Co. (St. Louis, MO, USA). Hydrogen peroxide (H_2_O_2_) solution (30% purified) was purchased from Merck (Darmstadt, Germany). The primary antibodies for Nrf2, HO-1, p-Akt, Akt, and β-actin, as well as all secondary antibodies and tin protoporphyrin IX (SnPP), were obtained from Santa Cruz Biotechnology (Santa Cruz, CA, USA). The primary antibodies for Keap1 and PCNA were supplied by Genetex (Irvine, CA, USA). Rabbit anti-tyrosine hydroxylase (TH), rabbit anti-dopamine transporter (DAT) and polyvinylidene fluoride were obtained from Millipore (Marlborough, MA, USA). Trolox was provided by Cayman Chemical (Ann Arbor, MI, USA). The ARE-binding site-luciferase construct was a generous gift from Prof. Young-Joon Surh (Seoul National University, Seoul, Korea). All other chemicals were purchased from Sigma-Aldrich Co. unless otherwise specified.

### 2.2 Cell culture

PC12 rat pheochromocytoma cell line was purchased from the American Type Culture Collection (Manassas, VA, USA). PC12 cells were cultured in Ham's F-12K medium, supplemented with 15% HS, 2.5% FBS. Cells were maintained in the presence of 100 U/ml penicillin and 100 μg/ml streptomycin at 37 °C in a humidified atmosphere of 5% CO_2_ and 95% air.

### 2.3 Measurement of cell viability

The cytoprotective effect of ECN on H_2_O_2_- or 6-OHDA-induced PC12 cells was measured with an MTT-based colorimetric assay. In brief, cells were treated with indicated concentrations of ECN before exposure to H_2_O_2_ (500 μM) or 6-OHDA (250 μM) for 24 h. MTT solution was added at the end of the treatment to the cell culture media at 0.5 mg/ml final concentration and incubated for 2 h at 37 °C in the dark. The absorbance at 595 nm was determined with an EMax® microplate reader (Molecular Devices, Sunnyvale, CA, USA).

### 2.4 Western blot analysis

Total cell lysates were prepared using the lysis buffer previously described [14]. To prepare cytosolic and nuclear extracts, cells were collected and washed with PBS. Cells were resuspended in the lysis buffer (10 mM HEPES [pH 7.9], 10 mM KCl, 0.1 mM EDTA, 0.1 mM EGTA, 1 mM DTT, 1 mM PMSF, and a protease inhibitor cocktail), incubated on ice for 15 min, and then 10% of NP-40 was added. The mixture was vortexed for 10 s and centrifuged at 15,000 rpm for 5 min, with this supernatant containing the cytoplasmic fraction. The nuclear pellets were resuspended in nuclear extraction buffer (20 mM HEPES [pH 7.9], 400 mM NaCl, 1 mM EDTA, 1 mM EGTA, 1 mM DTT, 0.5 mM PMSF, and a protease inhibitor cocktail) for 1 h on ice with vortexing at 10 min intervals and then centrifuged at 15,000 rpm for 10 min. The protein concentration was quantified using the Bradford protein assay (Bio-Rad Laboratories, Richmond, CA, USA). Equal amounts of protein (30 μg) were loaded on 8% of SDS polyacrylamide gels and transferred to nitrocellulose membranes. The membranes were blocked by 5% bovine serum albumin in T-BST buffer (20 mM Tris, 137 mM NaCl, 0.1% Tween 20, pH 7.6) and incubated with primary antibodies overnight at 4 °C. After washing, the membranes were incubated with horseradish peroxidase-conjugated secondary antibodies for 1 h at room temperature. The immunoblots were detected with EZ-Western detection kit (DoGEN, Seoul, Korea). The values above the figures represent the relative density of the bands normalized to that for β-actin or PCNA.

### 2.5 Quantitative real-time reverse transcriptase polymerase chain reaction (qRT-PCR)

Total RNA was isolated using the Trizol reagent kit (Invitrogen, Carlsbad, CA, USA). Both the quantity and purity of RNA were measured using the Nanodrop spectrophotometer (Thermo Scientific, Wilmington, DE, USA). Total RNA (1 μg) was synthesized into cDNA using the amfiRivert Platinum cDNA Synthesis Master Mix (GenDepot, Barker, TX, USA), in accordance with the manufacturer's instructions. PCR amplification of Nrf2, HO-1, and β-actin genes was performed using forward and reverse primers and a SYBR Green working solution (iTaq™ Universal SYBR Green Supermix, Bio-Rad, Hercules, CA, USA) with an Applied Biosystems 7300 real-time PCR system and software (Applied Biosystems, Carlsbad, CA, USA). The following primers were used: Nrf2, 5′-CTC GCT GGA AAA AGA AGT G-3′ (sense) and 5′-CCG TCC AGG AGT TCA GAG G-3′ (antisense); HO-1, 5′-CAC GCA TAT ACC CGC TAC CT-3′ (sense) and 5′-CCA GAG TGT TCA TTC GAG A-3′ (antisense).

### 2.6 ARE-luciferase assay

PC12 cells were co-transfected with 0.5 μg of pRL-CMV control vector (Promega, Madison, WI, USA) and 5 μg of ARE-luciferase plasmid using iN-fect™ transfection reagent (iNtRON Biotechnology, Seongnam, Korea), according to the manufacturer's instructions. After 24 h of transfection, the cells were treated with ECN for an additional 6 h or 24 h. The luciferase activity was performed using a dual luciferase assay kit (Promega, Madison, WI, USA).

### 2.7 Transient transfection of small interfering RNA

The cells, which were 60–80% confluent, were transfected with 100 nM scrambled, small interfering RNA (siRNA), Nrf2 and HO-1 siRNA (Bioneer, Daejeon, Korea) using Lipofectamine RNAiMAX (Invitrogen, Carlsbad, CA, USA) according to the manufacturer's instructions. After a 48 h transfection, cells were treated with ECN or other chemicals and then used for subsequent experiments.

### 2.8 Measurement of ROS accumulation

PC12 cells were seeded into 96-well plates (1 × 10^4^ cells/well) for 24 h, and then the medium was changed to serum-free medium. The cells were pretreated with ECN (2.5, 5, or 10 μM) for different times (10, 30, 60, and 120 min) or NAC (5 mM for 1 h) and incubated with 50 μM DCF-DA for 30 min at 37 °C. Fluorescence of DCF was detected by a multimode microplate reader (SpectraMax M5, Molecular Devices, Sunnyvale, CA, USA) at excitation and emission wavelengths of 485 and 538 nm, respectively.

### 2.9 Animals and surgery procedure

Male ICR mice (8 weeks old, 30–35 g) were purchased from Daehan Biolink Co., Ltd. (Eumseong, Korea). All animals were housed at a constant temperature of 23 ± 1 °C and a relative humidity of 60 ± 10% in a 12 h light/dark cycle. Animal treatment and maintenance were carried out in accordance with the Principle of Laboratory Animal Care (NIH publication No. 85–23, revised 1985) and the Animal Care and Use Guidelines of Kyung Hee University. All animal studies were performed according to the approved guidelines of the Institutional Animal Care and Use Committee of Kyung Hee University (approval ID: KHUASP(SE)-16-128).

Mice were anesthetized by an intraperitoneal injection of TBE (312.5 mg/kg body weight) and fixed into a stereotaxic apparatus (myNeuroLab, St. Louis, MO, USA). Each mouse received a unilateral injection of a 2 μl vehicle (saline with 0.1% (w/v) ascorbic acid, for control group) or 6-OHDA (16 μg/2 μl in 0.1% ascorbic acid-saline) into the right striatum (ST) (coordinates related to bregma in mm: anterior–posterior = 0.5, medial–lateral = 2.0, dorsal-ventral = – 3.0). Surgery was performed one day after the last drug administration.

### 2.10 Drug administration

The mice were divided into four groups, with six mice for each group: (1) sham group (sham-operated plus intraperitoneally saline-treated group); (2) ECN 5 mg/kg group (sham-operated plus intraperitoneally ECN 5 mg/kg treated group); (3) 6-OHDA group (6-OHDA-lesioned plus intraperitoneally saline-treated group); (4) 6-OHDA + ECN 5 mg/kg group (6-OHDA-lesioned plus intraperitoneally ECN 5 mg/kg treated group). ECN dissolved in saline was administered to the mice once per day for seven consecutive days.

### 2.11 Rotarod test

The rotarod test was conducted as described previously [15], with modifications on the 14th day after 6-OHDA injection. The mice were trained on the rotarod apparatus (diameter 3 cm; Ugo Basile, Collegeville, PA, USA) at a fixed speed of 12 rpm for 180 s. After training twice, mice were tested at a rotation speed of 17 rpm. The time each mouse remained on the rotating bar before falling was recorded with three trials, for a maximum of 180 s per trial. Data shown are the mean time spent on the rotarod over three test trials.

### 2.12 APO-induced rotation test

The rotation of all the mice induced by APO was tested on the 15th day after 6-OHDA lesion. Mice were placed in a hemispherical clear plastic bowl with a diameter of 40 cm and 360° turns in the direction opposite to the lesion (contralateral rotation) were counted for 25 min after subcutaneous injection of APO (4 mg/kg). Results were expressed as contralateral turns per 25 min.

### 2.13 Immunohistochemistry

After the completion of all behavioral testing, mice were transcardially perfused with 0.05 M PBS and then fixed with cold 4% PFA in a 0.1 M phosphate buffer. Brains were postfixed in a 0.1 M phosphate buffer containing 4% PFA overnight at 4 °C and then cryopreserved in 0.05 M PBS containing 30% sucrose. Frozen brains were cut into 30 µm thick coronal sections on a freezing microtome (Leica, Wetzlar, Germany) and stored in cryoprotectant (25% ethylene glycol, 25% glycerol, and 0.05 M phosphate buffer) at 4 °C until use. Free-floating sections were treated with H_2_O_2_ for 15 min, and then incubated with a rabbit anti-TH (1:1000 dilution) for SN and ST tissues or anti-DAT antibodies (1:500 dilution) for ST tissues overnight at 4 °C in PBS containing 0.3% Triton X-100 and normal goat serum. After washing with PBS, the sections were incubated with a biotinylated anti-rabbit IgG (1:200 dilution) for 90 min, followed by incubation with an avidin–biotin complex mixture (1:100 dilution) for 1 h at room temperature. The reaction was visualized by incubating sections with DAB for 3 min. The sections were rinsed in PBS, mounted onto gelatin-coated slides, dehydrated, and coverslipped using the histomount medium. The optical density of TH or DAT-positive fibers in ST was analyzed at ×40 magnification and the number of TH-positive cells in SN counted at ×100 magnification using the ImageJ software (Bethesda, MD, USA). The images were obtained using a microscope (Olympus Microscope System BX51, Olympus, Tokyo, Japan).

### 2.14 Statistical analysis

The results represent the mean ± the standard error of the mean (S.E.M.) from three different experiments. Statistical analyses were performed with a one-way analysis of variance (ANOVA) with Tukey's and/or Bonferroni's multiple comparisons test using GraphPad Prism 5.0 software (San Diego, CA, USA). *P* values less than 0.05 were considered statistically significant.

## 3 Results

### 3.1 ECN exerts protective effects against H_2_O_2_- or 6-OHDA-induced injury in PC12 cells

**Fig. 1:**
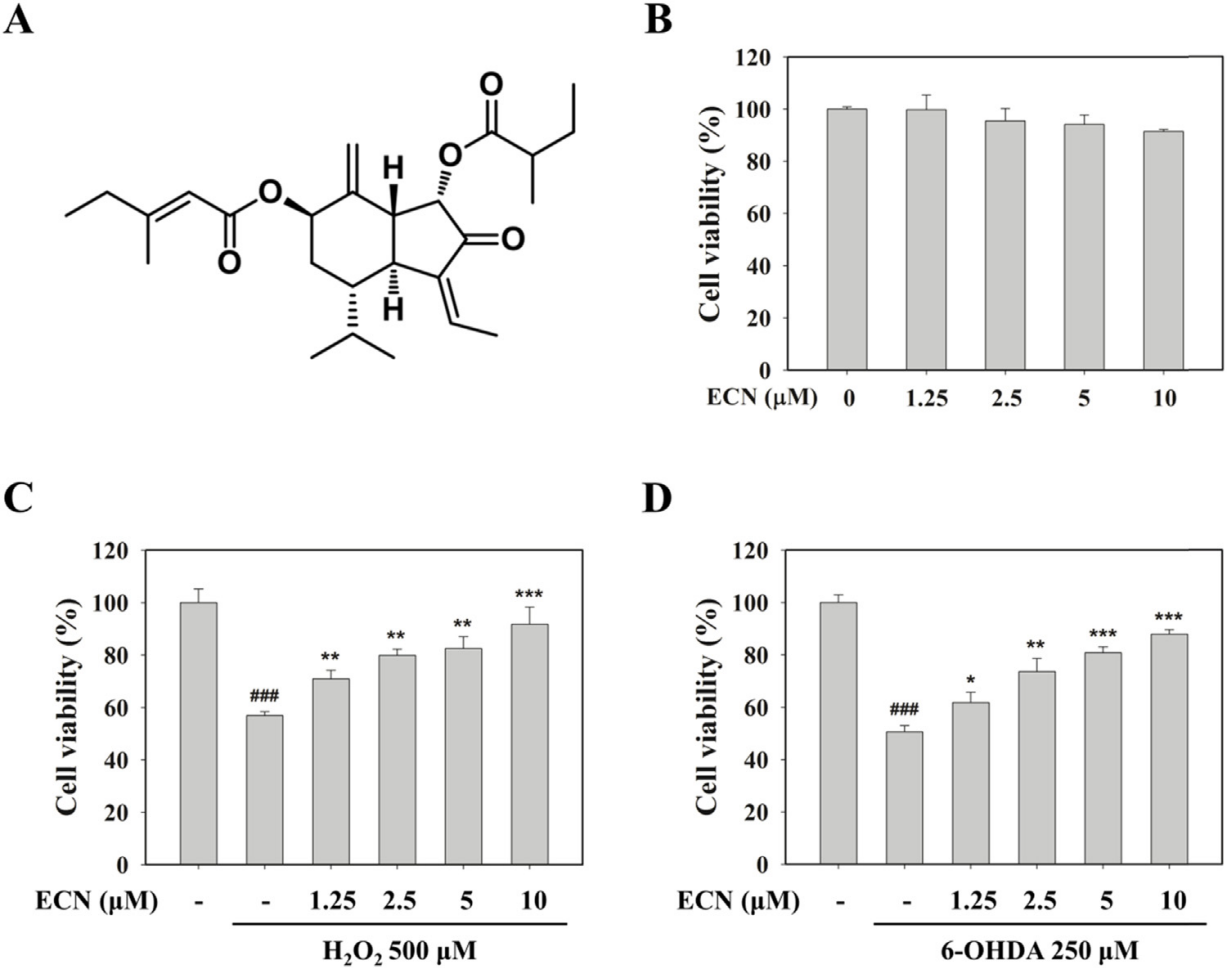
**Cytoprotective effects of ECN on H**_**2**_**O**_**2**_**- and 6-OHDA-induced cell injury in PC12 cells.** (A) The chemical structure of ECN isolated from the dried flower buds of *T. farfara*. (B) PC12 cells were treated with the indicated concentrations of ECN for 24 h and cell viability was determined by an MTT assay. (C and D) Cells were preincubated with various concentrations of ECN for 24 h and then exposed to 500 μM H_2_O_2_ (C) or 250 μM 6-OHDA (D) for an additional 24 h. ^###^*P* < 0.001 indicates a significant difference from the untreated control group. * *P* < 0.05, ** *P* < 0.01, and ***P < 0.001 indicate a significant difference compared with the H_2_O_2_- or 6-OHDA-only induced group.

To investigate whether ECN is cytoprotective against oxidative stress, we used H_2_O_2_ or 6-OHDA. ECN alone did not show any cytotoxicity at concentrations of up to 10 μM (Fig. 1B). Exposure to 500 μM H_2_O_2_ for 24 h decreased cell viability by 56.9 ± 1.5%, while pretreatment of PC12 cells with ECN 10 μM increased cell viability of up to 91.8 ± 6.6% (Fig. 1C). Incubation with 250 μM 6-OHDA for 24 h reduced cell viability to 50.6 ± 2.4%. However, pretreatment with 5 and 10 μM ECN significantly abolished (^***^P < 0.001, compared with the 6-OHDA group) the cytotoxic effect of 6-OHDA, increasing cell viability to 80.7 ± 2.3% and 87.9 ± 1.7%, respectively (Fig. 1D). These results suggest that ECN has protective effects against oxidative stress-induced cell damage in PC12 cells.

### 3.2 ECN up-regulates Nrf2 and HO-1 expression in PC12 cells at the transcriptional level

**Fig. 2:**
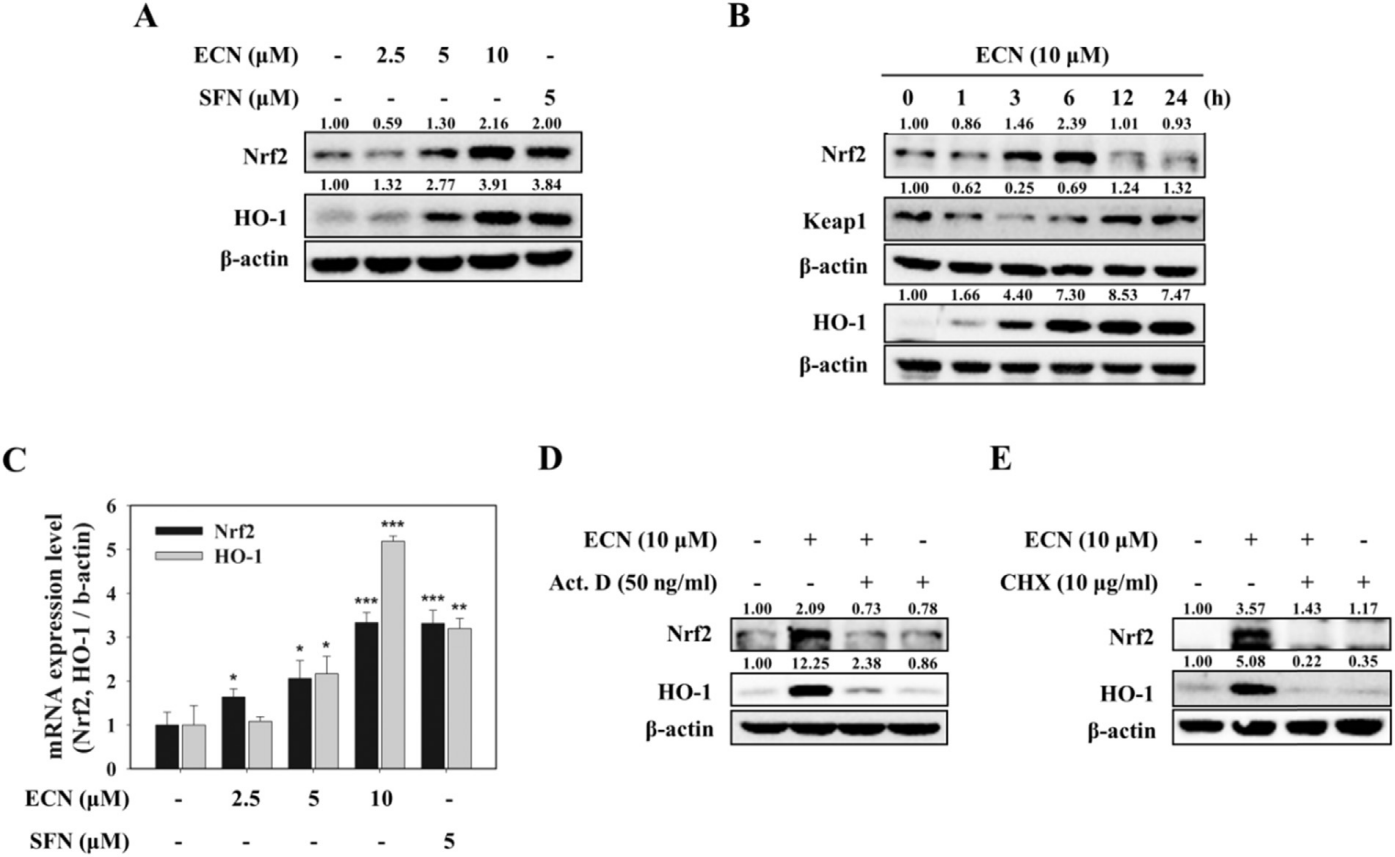
**Upregulation of Nrf2 and HO-1 expression at the transcriptional level in PC12 cells.** (A) PC12 cells were treated with the indicated concentrations of ECN for 6 h. Total cell lysates were prepared for Western blot analysis to determine Nrf2 and HO-1 protein levels. SFN was used as a positive control. (B) Cells were treated with 10 μM ECN for the indicated times. (C) Cells were pretreated with the indicated concentrations of ECN for 6 h and mRNA levels of Nrf2 and HO-1 were analyzed by real-time PCR. (D and E) Cells were treated with 10 μM ECN for 6 h in the presence of actinomycin D (Act. D, 50 ng/ml) or cycloheximide (CHX, 10 μg/ml).

The Nrf2 pathway is a valuable therapeutic target for neuroprotection [16]. Thus, we examined whether ECN activates the Nrf2/HO-1 pathway. PC12 cells were treated with various concentrations of ECN (2.5, 5, or 10 μM) for 6 h or with 10 μM ECN from 1 h to 24 h, and protein levels of Nrf2 and HO-1 were determined by a Western blot analysis. As shown in Fig. 2A, ECN treatment upregulated Nrf2 and HO-1 protein expressions dose dependently. Sulforaphane (SFN), a well-known Nrf2 activator, was used as a positive control. Time course study showed that Nrf2 protein reached a maximum level 6 h after 10 μM ECN treatment, whereas Keap1 protein changed in the opposite expression pattern to that of Nrf2. HO-1 protein expression increased 3 h after ECN treatment (Fig. 2B). We subsequently investigated whether the expression of both Nrf2 and HO-1 was regulated at the transcriptional level using real-time PCR. ECN treatment for 6 h induced a dose-dependent increase of both Nrf2 and HO-1 mRNA levels, showing 3.3- and 5.2-fold increase at 10 μM, respectively (Fig. 2C). In the presence of actinomycin D (Act. D, 50 ng/ml) or cycloheximide (CHX, 10 μg/ml), upregulations of Nrf2 and HO-1 by ECN were reduced, indicating that ECN regulated Nrf2 and HO-1 expression at the transcriptional and translational levels (Fig. 2D, E).

### 3.3 ECN-induced HO-1 expression is mediated through activation of Nrf2/ARE signaling

**Fig. 3:**
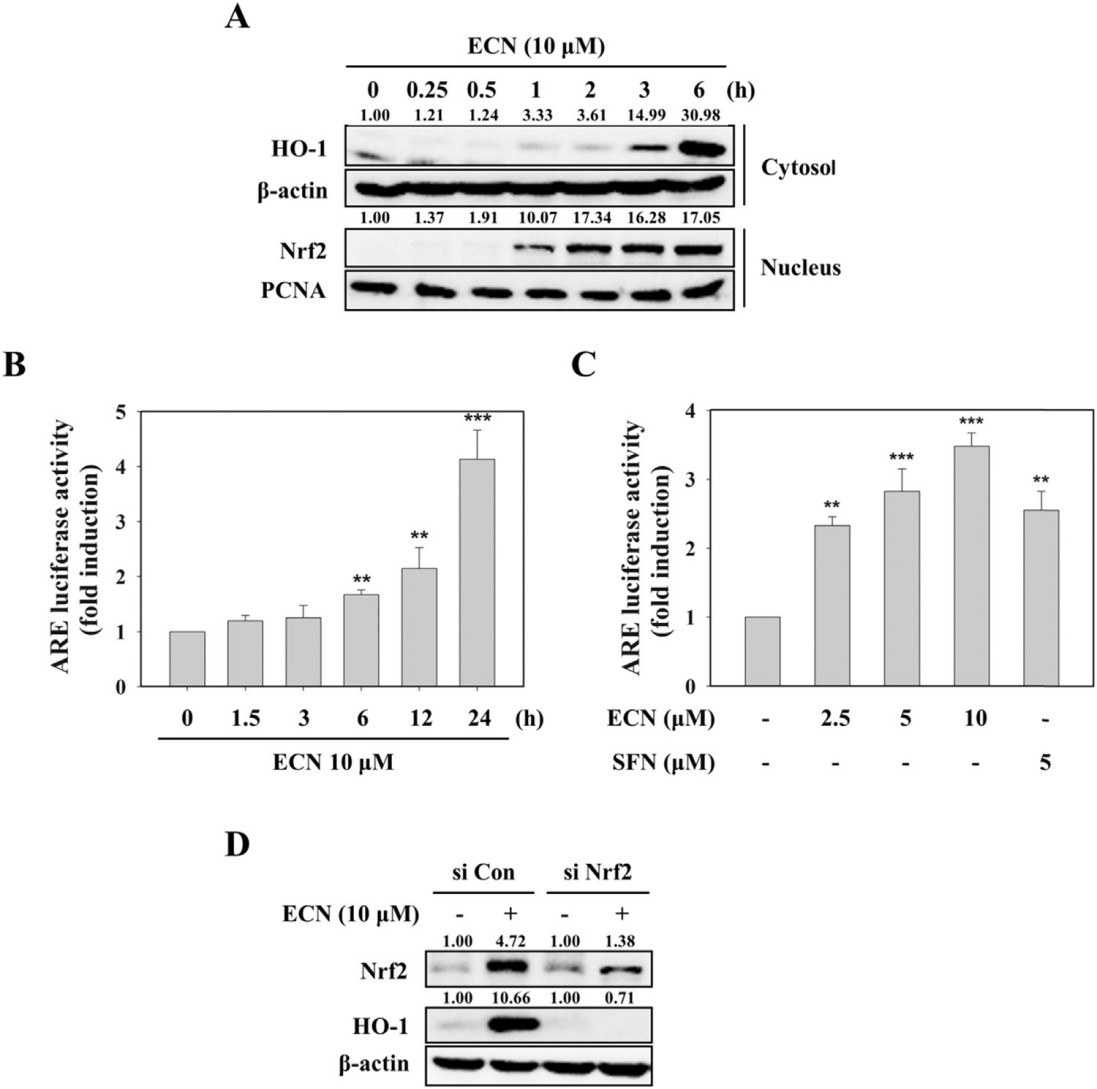
**Effects of ECN on the activation of ARE/Nrf2 signaling.** (A) PC12 cells were treated with 10 μM ECN for the indicated times. Nrf2 in the cytoplasm and nucleus and HO-1 in the cytoplasm were determined by Western blotting. (B and C) Cells were treated for the indicated times with 10 μM ECN (B) or 1 h with the indicated concentrations of ECN (C), and ARE-luciferase activity was measured. (D) Cells were transfected with 50 nM control siRNA (si Con) or Nrf2-targeted siRNA (si Nrf2) for 48 h and then treated with 10 μM ECN for 6 h.

When Nrf2/ARE pathway is activated by Nrf2 inducers, Nrf2 translocates to the nucleus and ultimately induces its downstream target genes such as HO-1 [17]. We identified whether ECN could enhance Nrf2 nuclear translocation and ARE-luciferase activity. ECN treatment increased the nuclear localization of Nrf2, as well as the expression of HO-1 in the cytoplasm (Fig. 3A). Treatment of ECN upregulated ARE-luciferase activity time and dose dependently (up to 4.1-fold and 3.5-fold, respectively) (Fig. 3B, C). To verify further whether ECN-induced HO-1 is mediated through activation of Nrf2, we transfected PC12 cells with siRNA targeting Nrf2. As shown in Fig. 3D, Nrf2 knockdown using siRNA blocked ECN-mediated HO-1 protein expression, suggesting that Nrf2 activation is crucial for ECN-induced HO-1 upregulation in PC12 cells.

### 3.4 ECN induces phosphorylation of Akt and may directly modify thiols of Keap1 for Nrf2 activation

**Fig. 4:**
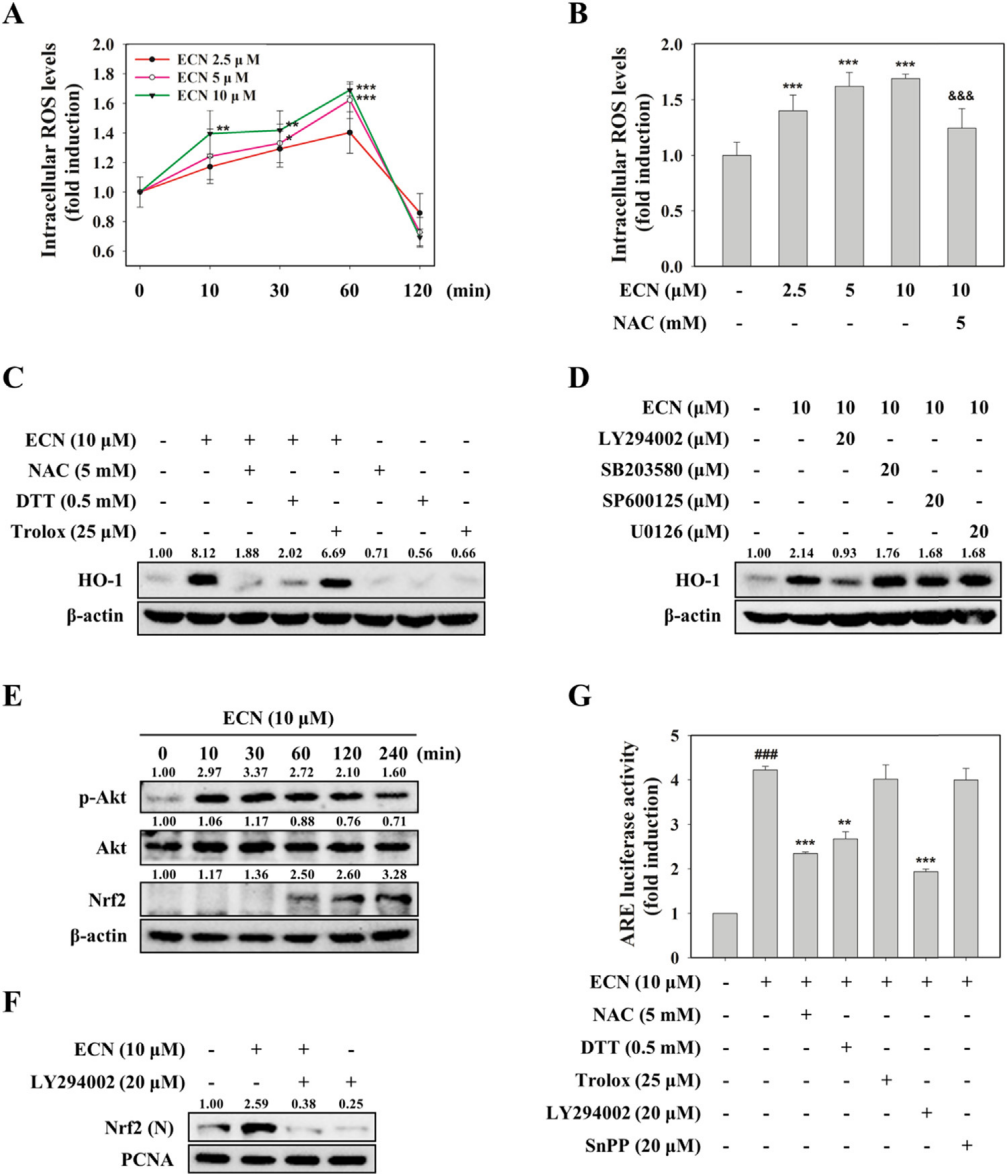
**Role of ROS and Akt in Nrf2 activation.** (A) PC12 cells were treated with ECN (2.5, 5, and 10 μM) for the indicated times and the levels of intracellular ROS were measured by DCFH-DA assay. * * *P* < 0.01 and ***P < 0.001 vs control (0 min). (B) Cells were treated with the indicated concentrations of ECN for 1 h. The antioxidant NAC served as a positive control. ***P < 0.001 vs control group; ^&&&^*P* < 0.001 vs ECN 10 μM treated group. (C and D) Cells were preincubated with various antioxidants (C) or kinase inhibitors (D) for 1 h before treatment with ECN (10 μM) for 6 h to determine HO-1 levels. (E) Cells were treated with ECN (10 μM) for indicated intervals and then p-Akt and Nrf2 protein levels were determined. (F) Cells were pretreated with LY294002 for 1 h and then treated with ECN for 1 h. Nuclear levels of Nrf2 were analyzed. (G) Cells were pretreated with various antioxidants or kinase inhibitors for 1 h and then incubated with ECN for 24 h. ^###^*P* < 0.001 vs control group; ** *P* < 0.01 and ***P < 0.001 vs ECN 10 μM treated group.

As it has been reported that mild oxidative or electrophilic stress can activate the Nrf2 pathway [18], we examined if ECN activated the Nrf2/HO-1 signaling pathway by producing ROS. When PC12 cells were incubated with ECN for indicated time intervals, intracellular ROS levels measured by DCF-DA assay were increased time dependently until 1 h, and then decreased 2 h after treatment with ECN (Fig. 4A). Preincubation with 5 mM NAC eliminated 10 μM ECN-caused ROS generation (Fig. 4B). To elucidate further the role of ROS in ECN-induced Nrf2 and HO-1 activation, we used NAC (5 mM) and DTT (0.5 mM) as thiol reducing agents and Trolox (25 μM) as a non-thiol reducing antioxidant. Pretreatment with NAC or DTT abrogated ECN-induced HO-1 expression and Nrf2 transcriptional activity. However, Trolox did not reverse the effect of ECN on HO-1 and Nrf2 activation (Fig. 4C, G). Next, we investigated whether MAPKs or Akt contributed to the activation of Nrf2/HO-1. Pharmacological inhibition of Akt with LY294002, but not that of p38 (SB203580), JNK (SP600125), and ERK (U0126), inhibited ECN-induced HO-1 expression in PC12 cells (Fig. 4D). We also identified that ECN activated Akt phosphorylation at ser473 as early as 10 min after treatment (Fig. 4E). Pretreatment with LY294002 abolished ECN-induced nuclear translocation of Nrf2 and ARE reporter gene activity (Fig. 4F, G). Collectively, these findings reveal that ECN may stabilize Nrf2 and upregulate Nrf2-mediated HO-1, by directly interacting with Keap1 and modifying critical cysteine thiols present in Keap1 rather than acting as a mild pro-oxidant. In addition, phosphorylation of Akt plays a role as an upstream signal in ECN-induced Nrf2/HO-1 activation.

### 3.5 Neuroprotective effect of ECN against oxidative stress is mediated by Nrf2/HO-1 signaling

**Fig. 5:**
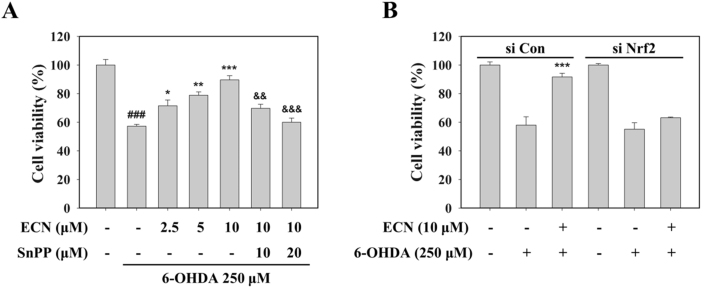
**Nrf2/HO-1 signaling-mediated neuroprotective effects of ECN.** (A) PC12 cells were pretreated with SnPP (10 or 20 μM) for 1 h before treatment with ECN (2.5, 5, and 10 μM) for 24 h and then exposed to 250 μM 6-OHDA for an additional 24 h. Cell viability was determined by the MTT assay. Data are presented as the mean ± SD. ^###^*P* < 0.001 indicates a significant difference from the 6-OHDA untreated control group; *P < 0.05, **P < 0.01, and ***P < 0.001 indicate a significant difference compared with the 6-OHDA-only exposed group; ^&&^*P* < 0.01 and ^&&&^*P* < 0.001 *versus* the 10 μM ECN plus 6-OHDA treated group. (B) Cells were transfected with 50 nM control siRNA (si Con) or Nrf2-targeted siRNA (si Nrf2) for 48 h and then treated with 10 μM ECN. After 24 h, cells were exposed to 250 μM 6-OHDA for an additional 24 h.

To determine whether the neuroprotective effect of ECN against oxidative stress was attributed to Nrf2/HO-1 signaling, we used SnPP, a HO-1 activity inhibitor, and a knockdown of Nrf2 by siRNA transfection. Pretreatment with SnPP dose-dependently reversed the protective effect of 10 μM ECN (Fig. 5A), and the Nrf2 siRNA transfection abolished the protective effect of ECN against 6-OHDA induced cell damage (Fig. 5B). Taken together, these results indicate that activation of the Nrf2/HO-1 pathway is required for the neuroprotection of ECN in PC12 cells.

### 3.6 ECN ameliorates 6-OHDA-induced motor impairments

**Fig. 6:**
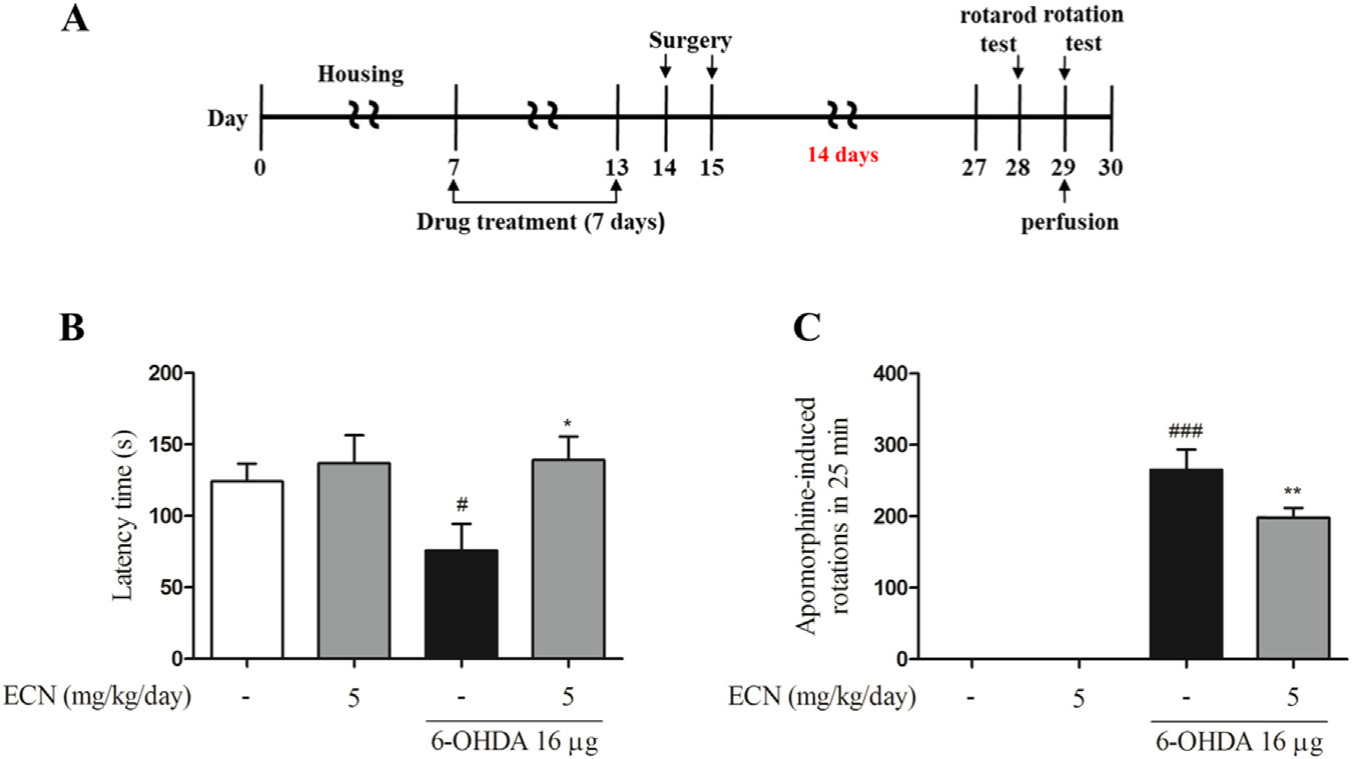
**Effects of ECN on 6-OHDA-induced motor impairment in mice.** (A) Schematic representation of the *in vivo* experimental design describing the treatment periods with 6-OHDA and ECN. ECN at 5 mg/kg dissolved in normal saline was administered for seven days. 6-OHDA was injected unilaterally *via* stereotaxic surgery in the right ST at one day after the last drug administration. (B) On the 14th day after 6-OHDA injection, latency time on the rotarod was tested. Data shown represent the three trial average time on the rotarod. (C) Contralateral rotations induced by APO were measured for 30 min 15 days after 6-OHDA lesion. ^#^*P* < 0.05 and ^###^*P* < 0.001 as compared with the saline-treated control group; * *P* < 0.05 and **P < 0.01 as compared with the 6-OHDA group.

To demonstrate whether ECN functions as a potent neuroprotective agent on an *in vivo* model, we next examined the effects of ECN on a 6-OHDA-induced mouse model (Fig. 6A). Two kinds of behavior tests, rotarod test and apomorphine (APO)-induced rotation test, were conducted. The results from the rotarod test showed that the 6-OHDA injection impaired performance (^*#*^*P* < 0.05, compared with the control group). This was measured by the length of time that mice stayed on the rotating rod. However, ECN administration (5 mg/kg/day, for seven consecutive days) before 6-OHDA injection ameliorated this impairment, (^*^P < 0.05) compared with the 6-OHDA-only group (Fig. 6B). In the APO-induced rotation test, 6-OHDA significantly increased the number of rotations in 25 min (^*###*^*P* < 0.001, compared with the vehicle control), whereas pretreatment with ECN at 5 mg/kg/day considerably decreased the number of rotations (^**^P < 0.01) compared with the 6-OHDA group (Fig. 6C). These results indicate that ECN effectively improves 6-OHDA-induced movement impairments.

### 3.7 ECN prevents 6-OHDA-induced dopaminergic neuronal damage in the ST and SN of the mouse brain

**Fig. 7:**
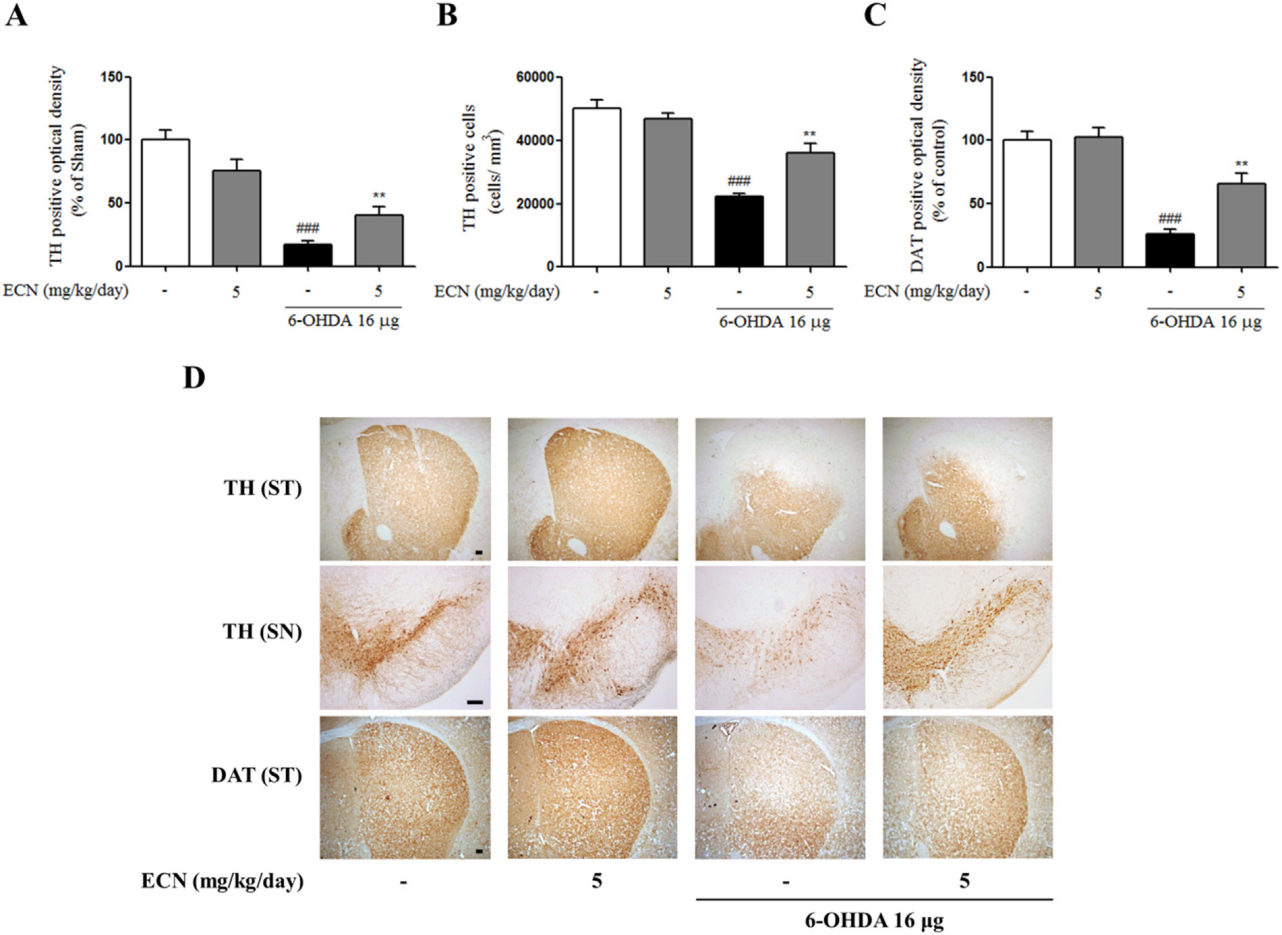
**Protective effects of ECN on 6-OHDA-induced dopaminergic neuronal loss in the ST and SN of mice.** (A) The optical density of TH-positive fibers in the ST was measured. (B) The numbers of TH-positive neurons in the SN were counted. (C) The optical density of DAT-positive fibers in the ST was measured. ^###^*P* < 0.001 as compared with the sham group; * * *P* < 0.01 as compared with the 6-OHDA group. (D) Representative immunostaining images are shown. Scale bar = 100 µm.

To confirm the neuroprotective effects of ECN against dopaminergic neuronal loss induced by 6-OHDA, we conducted immunostaining brain sections at the level of the SN and ST, for detecting TH and DAT—markers of dopaminergic neurons. Injection of 6-OHDA significantly reduced both TH-positive fibers and cells (^*###*^*P* < 0.001) when compared with the control, while pretreatment with ECN at 5 mg/kg ameliorated this loss in the ST and SN (^**^P < 0.01 compared with the 6-OHDA group) (Fig. 7A, B). A similar protective effect on DAT-positive fibers in the lesioned ST was also observed in the ECN-pretreated group (Fig. 7C). Taken together, these results obtained from immunohistochemistry support the hypothesis that ECN can protect dopaminergic neuronal loss against neurotoxicity induced by 6-OHDA. Representative images for these data are shown in Fig. 7D.

## 4 Discussion

The present study was designed to examine whether ECN provides Nrf2-mediated neuroprotective properties against oxidative stress. We demonstrated that ECN activated the Nrf2/HO-1 signaling pathway and had the ability to protect PC12 cells from damage caused by oxidative stress. The beneficial effects of ECN were reversed by the inhibition of Nrf2/HO-1, supporting the hypothesis that Nrf2 activation is responsible for the neuroprotective effects of ECN. This is the first study to reveal that ECN is a potent natural Nrf2 activator and has protective effects on 6-OHDA-induced *in vivo* model.

Because oxidative stress is closely associated with neuronal damage in neurodegenerative diseases [19], pharmacological agents that activate Nrf2 have been reported to be potent for the treatment of neurodegenerative diseases in different experimental models [10], [16]. Dimethyl fumarate (DMF), an approved drug for the treatment of multiple sclerosis, activates the Nrf2 pathway showing a protective effect against α-synucleinopathy toxicity in the murine model of PD [20]. A natural alkaloid, berberine, protected PC12 cells against 6-OHDA-induced neurotoxicity through activating the Nrf2/HO-1 signaling pathway and improved 6-OHDA-induced dopaminergic neuron loss and behavior movement deficiency in zebrafish, which supported the potency of berberine for the prevention and treatment of neurodegenerative diseases [21]. Here, we showed that ECN significantly activated Nrf2 and its target gene HO-1, which led to protective activity against oxidative stress in PC12 cells. Moreover, ECN alleviated motor deficits and dopaminergic neuronal damage in the 6-OHDA mouse model. Our results indicate that intraperitoneal administration of ECN has a protective effect against oxidative stress-induced neurotoxicity in the ST and SN of the mouse brain and PD-associated behavioral symptoms. Similar to other well-known Nrf2 activators such as curcumin and DMF [22], a structural feature of ECN that contains an α,β-unsaturated carbonyl moiety also supports the potential of ECN as a natural Nrf2 activator. Taken together, ECN may be a valuable pharmacological strategy for the treatment of oxidative stress-related neurodegenerative diseases, through activating the Nrf2 pathway.

Electrophiles and mild ROS have been demonstrated to induce Nrf2 activation and phase II enzymes [18], [23]. In addition, accumulated evidence supports that multiple protein kinases, including mitogen-activated protein kinases (MAPKs) and phosphatidylinositol-3-kinase (PI3K)/Akt, are associated with phosphorylation and nuclear accumulation of Nrf2 [24], [25]. For example, carnosol upregulated HO-1 through PI3K/Akt-dependent Nrf2 activation in PC12 cells [26]. Tussilagonone, one of the other sesquiterpenoids in *T. farfara*, phosphorylated MEK1/2 and ERK1/2 to induce Nrf2 nuclear accumulation and target genes showing cytoprotective effects in HepG2 cells [27]. Our data indicate that ECN with electrophilic property can directly modify Keap1 cysteine thiols, thereby leading to Nrf2 stabilization and HO-1 induction. This finding is in agreement with a previous report, which showed that guggulsterone with an electrophilic center activated Nrf2 through direct modification of critical cysteine residues in Keap1 [28]. Further study is required to clarify which cysteine residue is a direct target of ECN. We also investigated whether upstream kinases are responsible for ECN-induced Nrf2 activation. Pretreatment of cells with PI3K/Akt LY294002 inhibited ECN-induced nuclear accumulation of Nrf2, ARE-luciferase activity, and HO-1 expression, suggesting the essential role of the Akt pathway in regulating the Nrf2-ARE pathway by ECN.

The neurotoxin 6-OHDA *in vivo* model is widely used to study the therapeutic potential of drugs to treat PD [21]. However, the neurotoxin-based animal models of PD often fall short in replicating the true aspects of idiopathic PD, thus, the results of animal studies have failed to translate into success in clinical trials [29], [30], [31]. Despite these limitations, 6-OHDA model is a useful tool for identifying neuroprotective effects associated with oxidative stress because it generates reactive oxygen species and mimics the neuropathological and biochemical features of neurodegeneration in PD, such as motor and behavioral deficits and the loss of dopaminergic neurons [32], [33], [34]. ECN administration ameliorated 6-OHDA-induced motor impairment and hypokinesia in the rotarod test and APO-induced rotation test. Because many studies have demonstrated a strong relationship between behavioral impairment and nigrostriatal dopaminergic neuronal loss [35], [36], [37], [38], [39], we investigated neuronal loss by measuring immunoreactivity of TH and DAT in the ST and SN of mice. TH, a rate-limiting enzyme of catecholamine biosynthesis, is a marker for dopaminergic neurons. In addition, DAT, a transmembrane transporter, is responsible for the re-uptake of extracellular dopamine into presynaptic neurons. Because PD is closely associated with dopamine metabolism, TH and DAT have been considered to be important markers and therapeutic targets for PD [39], [40], [41]. A study in human brain tissues supports this notion by showing that there were reductions in TH and DAT staining in the nigrostriatal area of PD patients [42]. In this study, intrastriatal injection of 6-OHDA induced the loss of TH- and DAT-positive neurites in the ST- and TH-positive cells in the SNpc, which is consistent with the previous study [43]. However, the ECN-treated group recovered the down-regulated TH- and DAT-positive neurons of 6-OHDA-induced mice. Thus, we report here that ECN-induced improvement of behavioral abnormalities in the mouse model is the result of a suppression of dopaminergic neuronal loss. Although further study is needed to evaluate whether ECN crosses the blood–brain barrier (BBB), several lines of evidence imply that bioavailability of ECN may make it pharmacologically effective *in vivo*. ECN, with a molecular weight of 430, has no ability to form hydrogen bonds with water according to the rules for hydrogen bonding [44], indicating that it possesses the physicochemical properties for BBB penetration [45]. Moreover, the estimated brain-to-plasma partition coefficient denoted by *K*_p,brain_ value of ECN is 16.1 by an already known prediction method [46], [47], which means the total concentration of ECN in human brain tissue is predicted to be 16.1 fold higher than that in plasma.

**Fig. 8:**
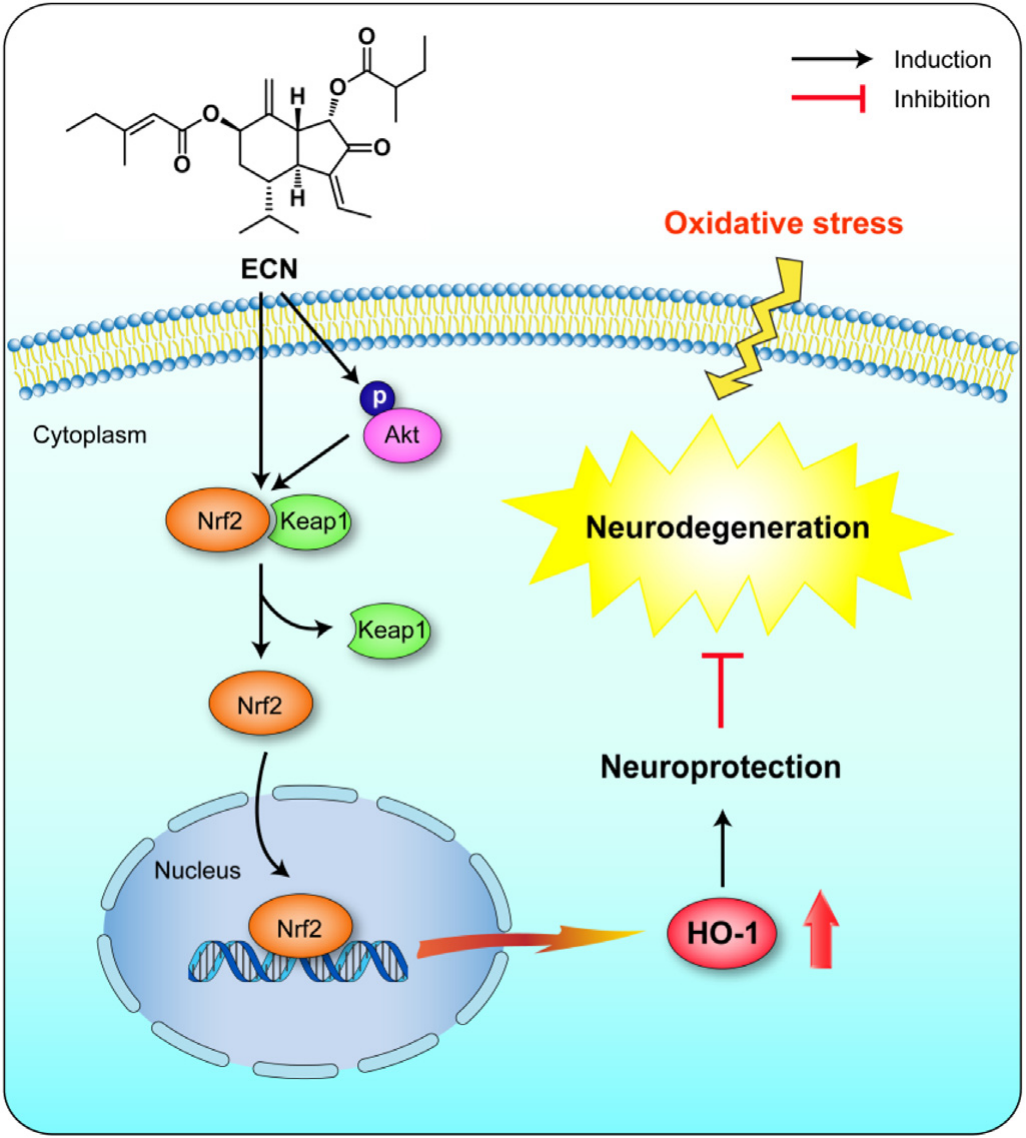
The proposed mechanism of Nrf2/ARE-mediated protective effects of ECN against oxidative stress-induced neurodegeneration.

In conclusion, the current study revealed that ECN, a sesquiterpenoid from the medicinal plant *T. farfara*, exhibited neuroprotective effects against oxidative stress-induced cell damage and dopaminergic neurodegeneration in mice. In addition, its mechanism of action through potential activation of the Nrf2/ARE signaling pathway has been elucidated (Fig. 8). These results suggest that targeting Nrf2 activation by a potent naturally occurring Nrf2 activator, ECN, is a promising therapeutic approach for the prevention or treatment of neurodegenerative conditions.

## References

[1] Barnham K.J., Masters C.L., Bush A.I. (2004). Neurodegenerative diseases and oxidative stress. Nat. Rev. Drug Discov..

[2] Lin M.T., Beal M.F. (2006). Mitochondrial dysfunction and oxidative stress in neurodegenerative diseases. Nature.

[3] Dias V., Junn E., Mouradian M.M. (2013). The role of oxidative stress in Parkinson's disease. J. Park. Dis..

[4] Cheignon C., Tomas M., Bonnefont-Rousselot D., Faller P., Hureau C., Collin F. (2017). Oxidative stress and the amyloid beta peptide in Alzheimer's disease. Redox Biol..

[5] Buendia I., Michalska P., Navarro E., Gameiro I., Egea J., León R. (2016). Nrf2–ARE pathway: an emerging target against oxidative stress and neuroinflammation in neurodegenerative diseases. Pharmacol. Ther..

[6] Joshi G., Johnson J.A. (2012). The Nrf2-ARE pathway: a valuable therapeutic target for the treatment of neurodegenerative diseases. Recent Pat CNS Drug Discov..

[7] Chen P.-C., Vargas M.R., Pani A.K., Smeyne R.J., Johnson D.A., Kan Y.W., Johnson J.A. (2009). Nrf2-mediated neuroprotection in the MPTP mouse model of Parkinson's disease: critical role for the astrocyte. Proc. Natl. Acad. Sci. USA.

[8] Satoh T., Okamoto S.-I., Cui J., Watanabe Y., Furuta K., Suzuki M., Tohyama K., Lipton S. (2006). Activation of the Keap1/Nrf2 pathway for neuroprotection by electrophillic phase II inducers. Proc. Natl. Acad. Sci. USA.

[9] Jazwa A., Cuadrado A. (2010). Targeting heme oxygenase-1 for neuroprotection and neuroinflammation in neurodegenerative diseases. Curr. Drug Targets.

[10] Kumar H., Kim I.-S., More S.V., Kim B.-W., Choi D.-K. (2014). Natural product-derived pharmacological modulators of Nrf2/ARE pathway for chronic diseases. Nat. Prod. Rep..

[11] Magesh S., Chen Y., Hu L. (2012). Small molecule modulators of Keap1‐Nrf2‐ARE pathway as potential preventive and therapeutic agents. Med. Res. Rev..

[12] Lim H.J., Dong G.-z., Lee H.J., Ryu J.-H. (2015). In vitro neuroprotective activity of sesquiterpenoids from the flower buds of Tussilago farfara. J. Enzym. Inhib. Med. Chem..

[13] Song K., Lee K.J., Kim Y.S. (2017). Development of an efficient fractionation method for the preparative separation of sesquiterpenoids from Tussilago farfara by counter-current chromatography. J. Chromatogr. A.

[14] Lee J., Kang U., Seo E.K., Kim Y.S. (2016). Heme oxygenase-1-mediated anti-inflammatory effects of tussilagonone on macrophages and 12-*O*-tetradecanoylphorbol-13-acetate-induced skin inflammation in mice. Int. Immunopharmacol..

[15] Choi J.G., Park G., Kim H.G., Oh D.-S., Kim H., Oh M.S. (2016). In vitro and in vivo neuroprotective effects of walnut (Juglandis semen) in models of Parkinson's disease. Int. J. Mol. Sci..

[16] Johnson D.A., Johnson J.A. (2015). Nrf2—a therapeutic target for the treatment of neurodegenerative diseases. Free Radic. Biol. Med..

[17] Zhang D.D. (2006). Mechanistic studies of the Nrf2-Keap1 signaling pathway. Drug Metab. Rev..

[18] Surh Y.-J. (2003). Cancer chemoprevention with dietary phytochemicals. Nat. Rev. Cancer.

[19] Calabrese V., Santoro A., Monti D., Crupi R., Di Paola R., Latteri S., Cuzzocrea S., Zappia M., Giordano J., Calabrese E.J. (2018). Aging and parkinson's disease: inflammaging, neuroinflammation and biological remodeling as key factors in pathogenesis. Free Radic. Biol. Med..

[20] Lastres-Becker I., García-Yagüe A.J., Scannevin R.H., Casarejos M.J., Kügler S., Rábano A., Cuadrado A. (2016). Repurposing the NRF2 activator dimethyl fumarate as therapy against synucleinopathy in Parkinson's disease. Antioxid. Redox Signal..

[21] Zhang C., Li C., Chen S., Li Z., Jia X., Wang K., Bao J., Liang Y., Wang X., Chen M. (2017). Berberine protects against 6-OHDA-induced neurotoxicity in PC12 cells and zebrafish through hormetic mechanisms involving PI3K/AKT/Bcl-2 and Nrf2/HO-1 pathways. Redox Biol..

[22] Wu R.P., Hayashi T., Cottam H.B., Jin G., Yao S., Wu C.C., Rosenbach M.D., Corr M., Schwab R.B., Carson D.A. (2010). Nrf2 responses and the therapeutic selectivity of electrophilic compounds in chronic lymphocytic leukemia. Proc. Natl. Acad. Sci. USA.

[23] Lee K.-M., Kang K., Lee S.B., Nho C.W. (2013). Nuclear factor-E2 (Nrf2) is regulated through the differential activation of ERK1/2 and PKC α/βII by Gymnasterkoreayne B. Cancer Lett..

[24] Surh Y.-J. (2012). Nrf2, an essential component of cellular stress response, as a potential target of hormetic phytochemicals. J. Food Drug Anal..

[25] Bryan H.K., Olayanju A., Goldring C.E., Park B.K. (2013). The Nrf2 cell defence pathway: Keap1-dependent and -independent mechanisms of regulation. Biochem. Pharmacol..

[26] Martin D., Rojo A.I., Salinas M., Diaz R., Gallardo G., Alam J., de Galarreta C.M.R., Cuadrado A. (2004). Regulation of heme oxygenase-1 expression through the phosphatidylinositol 3-kinase/Akt pathway and the Nrf2 transcription factor in response to the antioxidant phytochemical carnosol. J. Biol. Chem..

[27] Lee K.-M., Kwon T.Y., Kang U., Seo E.K., Yun J.H., Nho C.W., Kim Y.S. (2017). Tussilagonone-induced Nrf2 pathway activation protects HepG2 cells from oxidative injury. Food Chem. Toxicol..

[28] Almazari I., Park J.-M., Park S.-A., Suh J.-Y., Na H.-K., Cha Y.-N., Surh Y.-J. (2011). Guggulsterone induces heme oxygenase-1 expression through activation of Nrf2 in human mammary epithelial cells: PTEN as a putative target. Carcinogenesis.

[29] Athauda D., Foltynie T. (2015). The ongoing pursuit of neuroprotective therapies in Parkinson disease. Nat. Rev. Neurol..

[30] J. Potashkin, S. Blume, N. Runkle, Limitations of animal models of Parkinson’s disease, Parkinsons Dis. 2011. 10.4061/2011/658083 PMC3010694 21209719

[31] Bezard E., Yue Z., Kirik D., Spillantini M.G. (2013). Animal models of Parkinson's disease: limits and relevance to neuroprotection studies. Mov. Disord..

[32] Blesa J., Przedborski S. (2014). Parkinson's disease: animal models and dopaminergic cell vulnerability. Front. Neuroanat..

[33] Simola N., Morelli M., Carta A.R. (2007). The 6-hydroxydopamine model of Parkinson's disease. Neurotox. Res..

[34] Marin C., Aguilar E. (2011). In vivo 6-OHDA-induced neurodegeneration and nigral autophagic markers expression. Neurochem. Int..

[35] Dauer W., Przedborski S. (2003). Parkinson's disease: mechanisms and models. Neuron.

[36] Wang Q., Shin E.-J., Nguyen X.-K.T., Li Q., Bach J.-H., Bing G., Kim W.-K., Kim H.-C., Hong J.-S. (2012). Endogenous dynorphin protects against neurotoxin-elicited nigrostriatal dopaminergic neuron damage and motor deficits in mice. J. Neuroinflamm..

[37] Boix J., Padel T., Paul G. (2015). A partial lesion model of Parkinson's disease in mice–Characterization of a 6-OHDA-induced medial forebrain bundle lesion. Behav. Brain Res..

[38] Heuer A., Smith G.A., Lelos M.J., Lane E.L., Dunnett S.B. (2012). Unilateral nigrostriatal 6-hydroxydopamine lesions in mice I: motor impairments identify extent of dopamine depletion at three different lesion sites. Behav. Brain Res..

[39] Havrda M.C., Paolella B.R., Ward N.M., Holroyd K.B. (2013). Behavioral abnormalities and Parkinson's-like histological changes resulting from Id2 inactivation in mice. Dis. Model. Mech..

[40] Daubner S.C., Le T., Wang S. (2011). Tyrosine hydroxylase and regulation of dopamine synthesis. Arch. Biochem. Biophys..

[41] Vaughan R.A., Foster J.D. (2013). Mechanisms of dopamine transporter regulation in normal and disease states. Trends Pharmacol. Sci..

[42] Kordower J.H., Olanow C.W., Dodiya H.B., Chu Y., Beach T.G., Adler C.H., Halliday G.M., Bartus R.T. (2013). Disease duration and the integrity of the nigrostriatal system in Parkinson's disease. Brain.

[43] Penttinen A.M., Suleymanova I., Albert K., Anttila J., Voutilainen M.H., Airavaara M. (2016). Characterization of a new low‐dose 6‐hydroxydopamine model of Parkinson's disease in rat. J. Neurosci. Res..

[44] Pardridge W.M. (2012). Drug transport across the blood–brain barrier. J. Cereb. Blood Flow. Metab..

[45] Pajouhesh H., Lenz G.R. (2005). Medicinal chemical properties of successful central nervous system drugs. NeuroRx.

[46] Poulin P., Theil F.P. (2002). Prediction of pharmacokinetics prior to in vivo studies. 1. mechanism-based prediction of volume of distribution. J. Pharm. Sci..

[47] Poulin P., Theil F.P. (2002). Prediction of pharmacokinetics prior to in vivo studies. II. Generic physiologically based pharmacokinetic models of drug disposition. J. Pharm. Sci..

